# Variability of EEG electrode positions and their underlying brain regions: visualizing gel artifacts from a simultaneous EEG‐fMRI dataset

**DOI:** 10.1002/brb3.2476

**Published:** 2022-01-18

**Authors:** Catriona L. Scrivener, Arran T. Reader

**Affiliations:** ^1^ MRC Cognition and Brain Sciences Unit University of Cambridge Cambridge UK; ^2^ School of Philosophy, Psychology and Language Sciences University of Edinburgh Edinburgh UK; ^3^ Department of Psychology Faculty of Natural Sciences University of Stirling Stirling UK

**Keywords:** EEG‐fMRI, electrode positions, EEG cap | gel artifact, TMS neuro‐navigation

## Abstract

**Introduction:**

We investigated the between‐subject variability of EEG (electroencephalography) electrode placement from a simultaneously recorded EEG‐fMRI (functional magnetic resonance imaging) dataset.

**Methods:**

Neuro‐navigation software was used to localize electrode positions, made possible by the gel artifacts present in the structural magnetic resonance images. To assess variation in the brain regions directly underneath electrodes we used MNI coordinates, their associated Brodmann areas, and labels from the Harvard‐Oxford Cortical Atlas. We outline this relatively simple pipeline with accompanying analysis code.

**Results:**

In a sample of 20 participants, the mean standard deviation of electrode placement was 3.94 mm in *x*, 5.55 mm in *y*, and 7.17 mm in *z*, with the largest variation in parietal and occipital electrodes. In addition, the brain regions covered by electrode pairs were not always consistent; for example, the mean location of electrode PO7 was mapped to BA18 (secondary visual cortex), whereas PO8 was closer to BA19 (visual association cortex). Further, electrode C1 was mapped to BA4 (primary motor cortex), whereas C2 was closer to BA6 (premotor cortex).

**Conclusions:**

Overall, the results emphasize the variation in electrode positioning that can be found even in a fixed cap. This may be particularly important to consider when using EEG positioning systems to inform non‐invasive neurostimulation.

## Graphical abstract

We examined the variability of EEG (electroencephalography) electrode positions and their underlying brain regions by visualizing gel artifacts that arise on structural brain images in simultaneous EEG‐fMRI (functional magnetic resonance imaging). We observed that the scalp and cortical locations of parietal and occipital electrodes had the greatest between‐subject variability. We provide insight into the brain regions that lie underneath fixed cap electrodes in MNI space, with the notable finding that brain regions covered by electrode pairs are not always homologous.

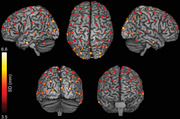

## 1 INTRODUCTION

Scalp electroencephalography (EEG) is one of the most frequently used neuroimaging methods, providing information about changes in electrical potential across the brain with high temporal resolution. Typical EEG setups measure activity across multiple points on the scalp. Electrodes are usually placed according to the international 10–20 system for around 21 channel recordings, 10–10 for between 64 and 85 channels, or 10–5 for high‐density caps of more than 300 channels (Jurcak et al., 2007; Oostenveld & Praamstra, 2001). These values refer to the distances between electrodes in relation to the total cap size (i.e., 20% of the total distance from the inion to the nasion) and aim to provide consistency across experiments. Electrodes are placed on the head of the participant with reference to anatomical landmarks such as the inion, nasion, and left and right pre‐auricular points, such that the central electrode Cz is approximately aligned with the vertex. Given careful placement of the electrode cap during experimental setup, experimenters assume that the electrode placement will be roughly consistent across participants. Further, when selecting a subset of electrodes for use in EEG analysis, we assume that they are in a similar position across participants and that we are comparing activation from similar regions of the brain.

Several studies have investigated electrode placement variations in the 10–20 (Atcherson et al., 2007; Herwig et al., 2003; Homan et al., 1987; Jack et al., 1990; Khosla et al., 1999; Lagerlund et al., 1993; Okamoto et al., 2004; Steinmetz et al., 1989; Towle et al., 1993) and 10‐10 (Koessler et al., 2009) systems. For example, Okamoto et al. (2004) recorded the normalized MNI and Talairach coordinates of electrode positions across 17 participants. From the 10–20 electrode layout used, FP1 and FP2 had the smallest deviation of around 5 mm in their MNI coordinates (reported across the *x*, *y*, and *z* dimensions), compared to the largest variation of roughly 10 mm identified in occipital electrodes O1 and O2. Each electrode position was also projected onto the cortical surface to provide an estimate of the underlying brain region. Using the mean location across all participants, the electrodes largely conformed to their intended positioning; for example, P3 and P4 projected to the superior parietal lobule and precuneus, and O1 and O2 projected to the occipital gyrus and cuneus. However, the electrodes commonly used to locate the motor cortex (C3 and C4), only projected to the precentral gyrus in 13% of cases. These results demonstrated the variation in location of electrodes in the 10–20 layout when collated across all participants and encourage some caution when assuming consistency in the underlying cortex.

Koessler et al. (2009) recorded the normalized Talairach coordinates of electrode positions projected onto the cortical surface using the 10‐10 electrode layout (rather than the 10–20) and therefore examined a greater number of electrodes than Okamoto et al. (2004). Across 16 participants, they reported a grand standard deviation of 4.6 mm in the *x* direction, 7.1 mm in *y*, and 7.8 mm in *z*, with variation across projected cortical positions. FP2 had the smallest global standard deviation of 67 mm^3^ and P1 had the largest of 548 mm^3^. Some electrodes projected to the same region consistently (FP1, FP2, O1, and O2), whereas others had larger variability (C6 and FC6). For example, FP1, FP2, FC1, and FC2 projected onto the superior frontal gyrus in 100% of participants, and O1 and O2 always projected onto the occipital gyrus (BA18: 81%, BA19: 19%). In comparison, most central and parietal electrodes projected onto four different Brodmann areas across participants; electrode P4 projected to BA39 (31%), 7 (25%), 40 (25%), 19 (19%), and electrode P8 projected to BA19 (56%), 37 (19%), 20 (12.5%), 39 (12.5%). Overall, variability in the underlying cortical regions was smallest for frontal and temporal electrodes, and greatest for central and parietal electrodes. This again suggests not only that the positions vary across participants, but that the consistency of these positions is electrode and region dependent.

Whilst these results may have important implications for making inferences from data derived from electrode positions, both Koessler et al. (2009) and Okamoto (2004) compared the location of manually positioned electrodes, without the aid of a cap with fixed locations. Therefore, errors in manual placement could have increased the variation in electrode location across participants. Atcherson et al. (2007) recorded the three‐dimensional locations of 15 electrodes fixed within a 72 channel Neuromedical Quick Cap. Despite the addition of an electrode cap, the electrode locations had standard deviations ranging from 3 mm to 12.7 mm in pre‐auricular‐nasion coordinates. In this case, the largest deviations occurred in M1 and M2, placed over the mastoids, as well as FPz (the most frontal central electrode) and Iz (the most posterior occipital electrode). The largest deviations therefore occurred in the electrodes around the edge of the cap, which could be explained by variations in participant skull shapes.

Overall, several studies have provided evidence against the assumption that a chosen electrode of interest will be proximally located to the same region of cortex across participants. This is perhaps not surprising, given the potential extent of between‐subject variability in the size and arrangement of the cerebral cortex. However, consistent placement of EEG electrodes is often assumed when their location is used to inform other methods. For example, the 10–20 and 10‐10 electrode layouts are regularly used to guide transcranial magnetic stimulation (TMS), where stimulation sites are chosen based on the position of specific electrodes such as those over the dorsolateral prefrontal cortex (Herwig et al., 2003). Structural or functional MRI‐guided TMS stimulation is often considered to be a more reliable technique (de Witte et al., 2018; Sack et al., 2009), and a recent meta‐analysis of repetitive TMS studies identified that MRI‐guided targets for stimulation were associated with increased disruptive effects of TMS (Beynel et al., 2019). However, in 2016 (the latest year included in the meta‐analysis), only 18% of studies used MRI‐guided TMS (Beynel et al., 2019). This constitutes a drop of 52% from studies between 2007 and 2013, suggesting a move back to older methods using EEG electrode position‐guided targeting, and the need for a re‐evaluation of the reliability of this approach.

The aim of this study was to further quantify the variability of EEG electrode positions in a commonly used research‐grade EEG cap layout. We took advantage of a pre‐existing neuroimaging dataset taken from a combined EEG and functional magnetic resonance imaging (fMRI) experiment, using 64 channel fixed electrode caps with a 10‐10 electrode layout (Scrivener et al., 2021). Whilst several groups have developed methods to recover EEG electrode positions from simultaneous EEG‐fMRI data using specific MRI acquisition methods (Butler et al., 2017) or reconstruction from acquired structural scans (Bhutada et al., 2020; Brinkmann et al., 1998; de Munck et al., 2012; Jurcak et al., 2005; Koessler et al., 2008; Kozinska et al., 2001; Lamm et al., 2001; Marino et al., 2016; Silva et al., 2016; Whalen et al., 2008), these approaches often require methods and toolboxes that are not yet widely used. As such, we additionally highlight a novel and simple way of projecting electrode locations to the cortical surface using electrode gel artifacts (that appear on the MR image underlying electrode positions) and commercially available equipment. We also provide the code to reproduce our results, or to apply to separate datasets.

This pipeline uses a stereotactic neuro‐navigation system in which electrode gel artifacts can be visualized using a scalp reconstruction function, facilitating localization of the electrode positions on the skull of each participant. These locations can then be projected onto the cortical surface. Using this method, we report the standard deviation of electrode positions on the skull and on the cortical surface, as well as the variability of underlying brain regions. As far as we are aware, electrode gel artifacts have not yet been used to provide a comprehensive assessment of EEG electrode position variability, either on the skull or the cortical surface, despite the fact they provide a simple method of localizing brain regions under the cap.

## 2 MATERIALS AND METHODS

We used 20 structural scans collected for a previously reported EEG‐fMRI experiment (Scrivener et al., 2021), for which the data are available at https://osf.io/w6bh3/. MRI data were recorded on a 3.0‐T Siemens Prisma scanner using a 64 channel head coil with participants lying in a head‐first supine position (3D MPRAGE; TE 2.37 ms; TR 1800 ms; flip angle 80 degrees; voxel size 0.98 × 0.98 mm; slice thickness 0.85 mm; slices per slab 208; FOV read 250 mm; ascending acquisition; phase encoding direction anterior to posterior). EEG data were recorded using a 64 channel fixed electrode cap (BrainAmp MR, Brain Products GmbH, Gilching, Germany). The secondary data for the current article, as well as MATLAB scripts used to analyze the data, are freely available at https://osf.io/853kw/. Participants in the original study consented for their data to be shared anonymously, and so only the defaced structural scans are freely available for download.

### 2.1 Electrode localization

**FIGURE 1:**
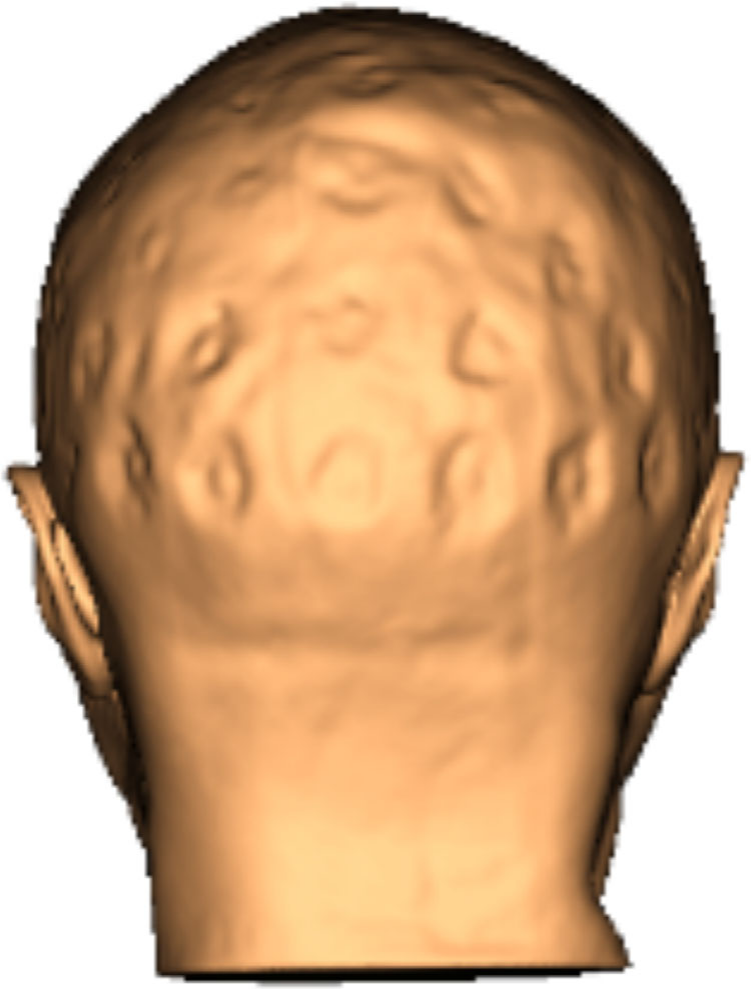
Gel artifacts visualized on the skull in Brainsight

Electrode positions were localized by author ATR using Brainsight 2.3.11 (Rogue Research Inc., Montreal, QC, Canada). The skin was reconstructed from the structural MRI scan to visualize electrode gel artifacts (Figure 1). Electrode positions were marked by placing targets onto the center of the gel artifacts, orthogonal to the skin. If a gel artifact was not clearly visible, the location of the electrode was inferred based on the surrounding electrode positions (18 across all participants, and never more than five in a single participant). The positions were independently checked by author CLS, and in cases of disagreement (nine electrodes across five participants) a consensus was met.

The electrode positions were then translated onto the underlying cortical surface. To do this we projected the targets to a curvilinear brain reconstruction (created using default parameters: slice spacing = 2 mm, end depth = 16 mm, peel depth = 0 mm) using the ‘‘snap to’’ function. Target positions (*xyz*) on the scalp and the curvilinear brain were exported as .txt files using the Brainsight review function.

### 2.2 Data analysis

The electrode positions on the scalp and cortex for each participant were translated into MNI space, using the affine transformation matrix generated by the SPM12 normalize function. This matrix provides the transformation needed to move from subject space to MNI space and allows for comparison across participants. To assess the variability of electrode positions, we calculated the mean and standard deviation of the location across participants for each electrode. This was calculated separately for scalp and cortical coordinates. Given that we had a recording of the cap size for most participants, we also report the locations separately for each cap size.

The brain regions at each electrode location were labeled using AtlasQuery in FSL and the Harvard‐Oxford Cortical Atlas (Desikan et al., 2006; Frazier et al., 2005; Goldstein et al., 2007; Makris et al., 2006), allowing us to visualize the consistency of brain regions underlying each electrode. For each electrode in each participant, we took the region with the highest probability reported by the atlas. We then calculated the regions reported for each electrode across all participants as a percentage. If multiple brain regions were reported with the same (highest) probability in an electrode for a single participant, we excluded that participant for the calculation of that electrode's underlying region. We also excluded electrodes from calculation if the atlas was not able to generate a label. Percentages were calculated based on the number of usable participants for each electrode (mean ± SD, participants = 17 ± 3). We also used BioImage Suite (https://bioimagesuiteweb.github.io/webapp/) to locate the Brodmann area associated with the mean coordinates of each electrode, to supplement this information.

The scripts to reproduce these results are freely available at https://osf.io/853kw/, which can also be used on independent data. To do this, researchers should save their electrode locations into a .txt file per participant, and provide a matrix describing the transformation from subject space to MNI space (e.g., as provided by the SPM normalize function). The MATLAB script provided will extract the locations given in the .txt file, save them into a results structure, calculate summary statistics, save the results into a .csv file, and save a nifti file for each participant with the locations plotted in MNI space. An additional Bash script is provided to pass each electrode coordinate to AtlasQuery in FSL and save the output into a .txt file.

## 3 RESULTS

### 3.1 Scalp locations

The mean electrode locations across participants can be found in Table 1. The skewness of these locations is reported in Supplementary Table S1. Overall, we found a grand standard deviation of 3.94 mm in *x*, 5.55 mm in *y*, and 7.17 mm in *z*. The five electrodes with the smallest overall deviation (mean SD = 4.47 mm) in *xyz* were mostly in frontal and central locations (F5, F7, FC5, FCz, FT7). The five electrodes with the largest overall deviation (mean SD = 6.78 mm) were in parietal and occipital locations (O1, P3, PO3. PO4, POz). There was no visible relationship between cap size and scalp position variability (Table 2).

**TABLE 1:** Mean and standard deviation of the MNI locations for each electrode, presented separately at the scalp and on the cortex

Electrode	Mean MNI coordinates in mm (SD)					
	Scalp			Cortex		
	*x*	*y*	*z*	*x*	*y*	*z*
AF3	−31.55 (4.56)	65.1 (4.78)	44.6 (5.99)	−25.41 (3.99)	54.96 (4.57)	36.6 (5.37)
AF4	34.61 (4.76)	65.6 (4.94)	42.92 (7.92)	28.06 (4.42)	55.43 (4.28)	35.32 (6.42)
AF7	−53.89 (3.69)	59.55 (4.31)	8.52 (6.68)	−45.43 (4.04)	52.57 (3.71)	7.20 (6.01)
AF8	55.41 (4.14)	59.76 (4.68)	10.20 (8.88)	46.40 (3.81)	52.38 (4.23)	8.78 (8.23)
AFZ	0.50 (4.55)	70.36 (4.65)	50.59 (7.57)	0.63 (3.84)	57.98 (4.52)	41.08 (6.31)
C1	−31.55 (4.94)	−21.59 (7.43)	92.04 (3.41)	−25.56 (4.77)	−23.82 (6.77)	75.26 (2.61)
C2	28.95 (5.68)	−21.98 (7.47)	94.55 (3.36)	23.84 (4.52)	−24.31 (6.80)	78.00 (3.21)
C3	−59.99 (4.12)	−19.25 (7.40)	70.68 (4.65)	−50.88 (4.53)	−21.18 (6.81)	59.95 (3.47)
C4	58.97 (4.73)	−21.28 (6.98)	73.84 (5.65)	50.78 (4.61)	−23.18 (6.17)	63.58 (4.78)
C5	−76.99 (2.19)	−19.50 (6.26)	37.19 (5.91)	−66.15 (3.20)	−20.58 (5.71)	33.83 (5.35)
C6	77.83 (2.15)	−21.23 (5.94)	39.99 (8.02)	66.48 (3.11)	−21.77 (5.42)	36.16 (6.97)
CP1	−32.35 (5.32)	−51.50 (7.52)	89.76 (3.69)	−25.64 (4.41)	−48.14 (5.73)	71.46 (3.43)
CP2	29.61 (4.72)	−52.43 (7.98)	92.91 (3.01)	24.46 (3.97)	−49.71 (6.28)	75.38 (3.28)
CP3	−59.90 (4.74)	−49.84 (7.27)	69.31 (5.29)	−49.15 (4.81)	−47.78 (5.87)	58.42 (3.80)
CP4	56.54 (4.27)	−51.89 (7.07)	73.89 (5.87)	46.66 (4.34)	−48.71 (5.69)	62.98 (4.35)
CP5	−74.32 (2.58)	−49.33 (6.09)	37.02 (8.16)	−63.88 (3.49)	−47.19 (5.69)	33.95 (7.01)
CP6	73.44 (2.21)	−52.25 (5.56)	41.24 (7.95)	62.43 (3.13)	−49.07 (4.98)	37.65 (6.65)
CPZ	−1.60 (4.97)	−53.38 (7.80)	96.60 (2.90)	−0.73 (4.32)	−50.47 (6.59)	75.85 (3.31)
CZ	−1.09 (4.43)	−22.15 (7.70)	99.93 (2.66)	−0.47 (3.61)	−24.64 (6.88)	80.16 (3.89)
F1	−25.78 (4.61)	39.37 (6.27)	72.50 (4.61)	−20.48 (4.35)	32.36 (5.57)	59.68 (5.07)
F2	26.17 (4.41)	39.90 (6.01)	73.75 (5.62)	20.96 (3.78)	32.86 (5.37)	59.93 (5.04)
F3	−46.04 (3.84)	40.62 (6.37)	55.73 (5.58)	−38.23 (3.74)	34.47 (5.99)	46.92 (5.14)
F4	46.51 (4.77)	41.51 (5.70)	57.56 (7.15)	38.59 (4.31)	34.99 (5.03)	48.23 (5.93)
F5	−59.99 (2.80)	40.30 (4.91)	33.12 (6.10)	−50.82 (3.08)	35.00 (4.51)	28.66 (5.50)
F6	62.13 (3.71)	39.34 (5.05)	34.98 (7.61)	52.34 (3.67)	33.9 (4.35)	30.57 (6.51)
F7	−68.45 (2.60)	35.32 (4.52)	4.68 (5.56)	−56.68 (2.82)	30.5 (4.28)	4.33 (5.10)
F8	71.6 (3.03)	32.35 (5.26)	6.88 (8.00)	59.39 (3.04)	27.37 (4.67)	6.97 (6.98)
FC1	−29.94 (4.43)	10.07 (7.18)	84.65 (4.01)	−24.69 (4.32)	5.73 (6.43)	71.11 (3.45)
FC2	28.88 (4.51)	9.22 (6.47)	86.09 (4.06)	24.09 (4.01)	5.40 (6.14)	72.18 (3.55)
FC3	−53.84 (3.95)	10.76 (6.85)	65.67 (5.19)	−46.09 (4.07)	7.35 (6.76)	56.46 (4.20)
FC4	54.9 (4.41)	10.08 (5.90)	67.56 (5.81)	47.52 (4.25)	6.49 (5.70)	58.34 (5.18)
FC5	−71.6 (2.29)	11.83 (5.91)	34.94 (5.11)	−61.1 (2.74)	8.02 (5.28)	30.64 (5.07)
FC6	73.25 (3.46)	9.72 (4.52)	38.28 (8.03)	62.59 (3.44)	6.62 (4.52)	33.69 (7.09)
FCZ	0.02 (3.73)	11.38 (6.8)	91.62 (3.31)	0.41 (3.01)	6.77 (6.27)	75.19 (4.06)
FP1	−29.72 (5.00)	77.81 (2.42)	13.85 (7.37)	−24.54 (4.48)	66.41 (2.57)	11.97 (6.81)
FP2	29.87 (5.19)	78.59 (3.18)	14.59 (9.50)	25.25 (4.43)	66.62 (2.86)	12.19 (7.77)
FPZ	−0.36 (5.19)	83.18 (2.18)	16.67 (8.54)	−0.29 (4.56)	69.71 (2.39)	13.71 (7.35)
FT10	78.78 (1.80)	0.51 (5.48)	−32.1 (8.41)	60.47 (5.25)	−1.67 (5.63)	−31.19 (8.88)
FT7	−77.03 (2.10)	8.63 (5.19)	2.98 (6.14)	−63.49 (3.62)	5.75 (4.43)	3.02 (5.36)
FT8	79.96 (1.76)	5.11 (4.37)	5.06 (8.60)	66.54 (2.85)	2.58 (3.83)	4.93 (8.12)
FT9	−77.33 (2.65)	1.91 (5.8)	−31.28 (6.38)	−59.09 (6.18)	0.07 (5.75)	−30.94 (5.82)
FZ	0.52 (4.56)	43.05 (6.34)	77.99 (5.14)	0.88 (3.46)	34.43 (5.64)	62.21 (4.84)
O1	−31.42 (5.64)	−109.90 (3.25)	8.94 (11.50)	−27.11 (4.68)	−99.78 (3.60)	6.68 (10.44)
O2	26.35 (4.88)	−110.54 (2.94)	11.57 (11.31)	22.51 (4.30)	−100.07 (2.74)	8.94 (10.18)
OZ	−2.53 (5.50)	−114.61 (2.37)	11.90 (11.48)	−2.49 (4.97)	−102.67 (3.09)	9.10 (10.31)
P1	−31.55 (5.73)	−77.61 (6.82)	75.8 (6.59)	−25.9 (4.45)	−68.45 (5.63)	61.21 (4.51)
P2	24.99 (5.99)	−78.59 (6.65)	77.65 (6.53)	20.8 (5.18)	−69.28 (6.73)	64.82 (4.87)
P3	−52.00 (5.32)	−77.05 (6.86)	58.59 (8.51)	−42.75 (4.45)	−69.51 (6.00)	49.82 (5.89)
P4	47.39 (4.70)	−78.76 (6.04)	61.12 (7.89)	39.28 (4.64)	−70.63 (5.65)	52.38 (6.17)
P5	−63.26 (3.79)	−77.35 (5.62)	30.97 (9.72)	−53.84 (3.61)	−71.50 (5.22)	28.15 (8.21)
P6	60.40 (3.56)	−78.77 (5.00)	36.36 (9.56)	51.14 (3.76)	−71.81 (4.69)	32.06 (8.35)
P7	−69.63 (3.32)	−73.74 (4.90)	0.78 (10.60)	−59.17 (2.82)	−69.14 (4.46)	0.70 (10.07)
P8	67.72 (2.35)	−75.60 (4.69)	5.83 (9.92)	57.52 (2.74)	−70.05 (4.18)	4.98 (9.43)
PO3	−34.84 (5.56)	−98.67 (5.19)	41.77 (9.39)	−29.71 (4.39)	−88.53 (5.70)	35.13 (8.07)
PO4	29.24 (5.60)	−98.85 (4.95)	45.00 (9.87)	25.37 (4.62)	−88.73 (5.24)	38.16 (8.93)
PO7	−54.12 (4.43)	−93.91 (3.75)	4.79 (10.26)	−46.45 (3.26)	−86.74 (3.80)	3.87 (9.43)
PO8	50.17 (3.84)	−95.92 (4.15)	8.99 (10.35)	42.78 (4.03)	−88.05 (3.60)	7.87 (9.83)
POZ	−3.28 (5.69)	−101.06 (5.15)	50.6 (9.14)	−2.76 (4.79)	−90.20 (5.71)	42.12 (7.54)
PZ	−2.25 (6.16)	−80.03 (7.27)	79.64 (6.14)	−1.94 (5.31)	−69.12 (7.17)	66.04 (4.54)
T7	−81.12 (1.60)	−20.17 (5.89)	0.58 (7.98)	−69.72 (2.66)	−20.31 (5.20)	0.55 (7.35)
T8	83.15 (1.08)	−23.69 (6.09)	4.20 (9.23)	71.04 (2.95)	−23.53 (5.69)	3.60 (8.61)
TP7	−78.17 (1.68)	−49.26 (4.7)	0.60 (9.15)	−68.19 (2.19)	−47.06 (4.03)	0.35 (8.25)
TP8	78.51 (1.70)	−52.05 (5.25)	3.53 (8.75)	67.73 (2.45)	−48.93 (5.09)	2.77 (7.97)
TP9	−73.37 (2.16)	−54.91 (4.44)	−35.64 (8.25)	−57.93 (4.26)	−52.6 (3.11)	−33.16 (7.65)
TP10	73.87 (2.55)	−57.04 (4.38)	−33.84 (10.41)	58.93 (3.95)	−53.09 (3.59)	−31.33 (9.05)

**TABLE 2:** Mean standard deviation of the MNI locations at the scalp and cortex presented separately for each cap size. Note that most participants had a cap size of 56 cm, and therefore the distribution is unequal. The cap size for one participant was not recorded

Cap size (cm)	n	Mean standard deviation of MNI coordinates in mm					
		Scalp			Cortex		
		x	y	z	x	y	z
54	4	3.89	3.05	6.21	3.42	2.85	5.43
56	12	4.26	5.54	7.80	4.26	5.54	7.00
58	3	2.42	5.66	6.02	3.04	5.12	5.22
All	20	3.94	5.55	7.17	3.95	5.09	6.35

### 3.2 Cortex locations

**FIGURE 2:**
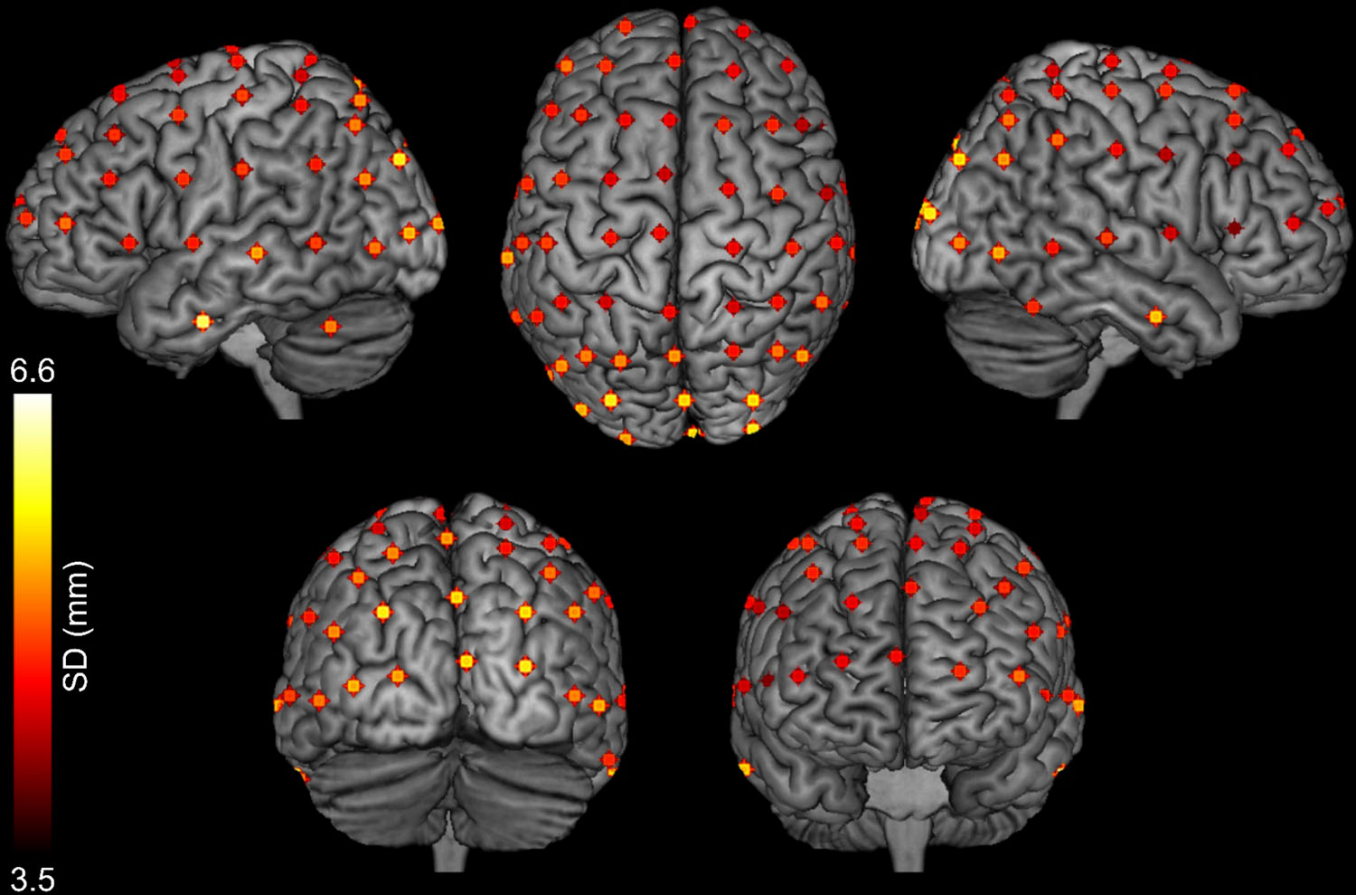
Mean projected cortex locations for each of 65 electrodes (including ground and reference) across 20 participants, displayed on an MNI template brain in MRICron. The standard deviation of each position is given by the color of the point, such that electrodes plotted in yellow had a higher standard deviation across participants than those plotted in red. For visualization purposes only, the mean co‐ordinate for each electrode was convolved with a 4 mm sphere

The mean cortical locations across participants are displayed on an MNI template brain in Figure 2, and can also be found in Table 1. The skewness of these locations is reported in Supplementary Table S1. Overall, we found a grand standard deviation of 3.95 mm in *x*, 5.09 mm in *y*, and 6.35 mm in *z*. The five electrodes with the smallest overall deviation (mean SD = 4.34 mm) in *xyz* were in frontal locations (F5, F7, FC5, FCz, FT7). The five electrodes with the largest overall deviation (mean SD = 6.25 mm) were in parietal and occipital areas (O1, Oz, PO3, PO4, FT10). There was no visible relationship between cap size and cortex position variability (Table 2). The cortical locations labeled using the Harvard‐Oxford Cortical Atlas and BioImage Suite can be found in Table 3.

**TABLE 3:** Electrode locations on the scalp labelled using AtlasLabel in FSL and the Harvard‐Oxford Cortical Structural atlas. For each electrode we calculated the percentage of participants reporting each anatomical label as the highest probability region. The closest Brodmann area for the mean MNI coordinate at each electrode projection is also detailed (generated with BioImage Suite)

Electrode	Percentage underlying brain regions	Brodmann area nearest mean electrode position
AF3	Frontal pole (100%)	Left BA9
AF4	Frontal pole (100%)	Right BA9
AF7	Frontal pole (100%)	Left BA10
AF8	Frontal pole (100%)	Right BA10
AFZ	Frontal pole (62.5%), SFG (37.5%)	Left BA9
C1	Precentral gyrus (71%), postcentral gyrus (29%)	Left BA4
C2	Precentral gyrus (87.5%), postcentral gyrus (12.5%)	Right BA6
C3	Postcentral gyrus (87.5%), precentral gyrus (12.5%)	Left BA1
C4	Postcentral gyrus (85%), precentral gyrus (15%)	Right BA1
C5	Postcentral gyrus (50%), anterior SMG (44.4%), precentral gyrus (5.6%)	Left BA40
C6	Anterior SMG (72%), postcentral gyrus (28%)	Right BA40
CP1	SPL (66.67%), postcentral gyrus (27.78%), superior LOC (5.56%)	Left BA7
CP2	SPL (80%), postcentral gyrus (13%), superior LOC (7%)	Right BA7
CP3	Posterior SMG (47%), SPL (27%), ANG (13%), postcentral gyrus (13%)	Left BA40
CP4	ANG (50%), SPL (42%), postcentral gyrus (8%)	Right BA7
CP5	Posterior SMG (61.11%), anterior SMG (16.67%), ANG (11.11%), posterior STG (5.56%), superior LOC (5.56%)	Left BA39
CP6	ANG (53%), posterior SMG (40%), superior LOC (7%)	Right BA39
CPZ	Postcentral gyrus (62.5%), precuneus cortex (18.75%), SPL (12.5%), superior LOC (6.25%)	Left BA7
CZ	Precentral gyrus (87%), postcentral gyrus (13%)	Right BA4
F1	SFG (71%), frontal pole (29%)	Left BA6/BA8
F2	SFG (77%), frontal pole (23%)	Right BA6/BA8
F3	MFG (64%), frontal pole (36%)	Left BA9
F4	Frontal pole (53%), MFG (47%)	Right BA9
F5	MFG (65%), frontal pole (35%)	Left BA9
F6	MFG (50%), frontal pole (50%)	Right BA9
F7	IFG (pars triangularis) (83%), frontal pole (11%), IFG (pars opercularis) (6%)	Left BA45
F8	IFG (pars triangularis) (66.67%), IFG (pars opercularis) (16.67%), MFG (5.56%), precentral gyrus (5.56%), frontal pole (5.56%)	Right BA45
FC1	SFG (93.75%), MFG (6.25%)	Left BA6
FC2	SFG (82%), precentral gyrus (18%)	Right BA6
FC3	MFG (89%), precentral gyrus (11%)	Left BA6
FC4	MFG (92%), precentral gyrus (8%)	Right BA6
FC5	Precentral gyrus (88%), MFG (6%), IFG (pars opercularis) (6%)	Left BA6
FC6	Precentral gyrus (81%), postcentral gyrus (13%), MFG (6%)	Right BA6
FCZ	JLC (66.7%), SFG (33.3%)	Left BA6
FP1	Frontal pole (100%)	Left BA10
FP2	Frontal pole (100%)	Right BA10
FPZ	Frontal pole (100%)	Left BA10
FT10	Anterior MTG (66.7%), posterior ITG (11.1%), temporal pole (11.1%), posterior MTG (5.6%), anterior ITG (5.6%)	Right BA21
FT7	Precentral gyrus (64.29%), temporal pole (14.29%), anterior STG (14.29%), IFG (pars opercularis) (7.14%)	Left BA44
FT8	Precentral gyrus (62.5%), anterior STG (25%), central opercular cortex (6.25%), posterior MTG (6.25%)	Right BA6
FT9	Anterior MTG (42%), temporal pole (37%), posterior MTG (16%), anterior ITG (5%)	Left BA38
FZ	SFG (100%)	Left BA6
O1	Occipital pole (100%)	Left BA18
O2	Occipital pole (100%)	Right BA18
OZ	Occipital pole (100%)	Left BA18
P1	Superior LOC (95%), SPL (5%)	Left BA7
P2	Superior LOC (100%)	Right BA7
P3	Superior LOC (95%), ANG (5%)	Left BA39
P4	Superior LOC (100%)	Right BA39
P5	Superior LOC (89.47%), inferior LOC (5.26%), ANG (5.26%)	Left BA39
P6	Superior LOC (100%)	Right BA39
P7	Inferior LOC (75%), superior LOC (20%), MTG (temporooccipital part) (5%)	Left BA19
P8	Inferior LOC (85%), superior LOC (10%), ANG (5%)	Right BA19
PO3	Superior LOC (65%), occipital pole (35%)	Left BA19
PO4	Occipital pole (58%), superior LOC (42%)	Right BA19
PO7	Inferior LOC (80%), superior LOC (15%), occipital pole (5%)	Left BA18
PO8	Occipital pole (33.33%), superior LOC (33.33%), inferior LOC (33.33%)	Right BA19
POZ	Cuneal cortex (37.5%), occipital pole (31.3%), superior LOC (25%), precuneus cortex (6.3%)	Left BA19
PZ	Precuneous cortex (60%), superior LOC (33.33%), SPL (6.66%)	Left BA7
T7	Posterior STG (72.2%), posterior MTG (16.6%), anterior STG (5.6%), anterior SMG (5.6%)	Left BA21
T8	Posterior STG (52.9%), posterior MTG (29.4%), planum temporale (5.9%), anterior SMG (5.9%), central opercular cortex (5.9%)	Right BA22
TP7	MTG (temporooccipital part) (73.7%), posterior SMG (15.8%), posterior MTG (10.5%)	Left BA21
TP8	MTG (temporooccipital part) (75%), ANG (20%), posterior SMG (5%)	Right BA37
TP9	ITG (temporooccipital part) (100%)	Not applicable (cerebellum)
TP10	ITG (temporooccipital part) (89%), MTG (temporooccipital part) (11%)	Not applicable (cerebellum)

*Abbreviations*: ANG, angular gyrus; IFG, inferior frontal gyrus; ITG, inferior temporal gyrus; JLC, juxtapositional lobule cortex (formerly supplementary motor cortex); LOC, lateral occipital cortex; MFG, middle frontal gyrus; MTG, middle temporal gyrus; SFG, superior frontal gyrus; SMG, supramarginal gyrus; SPL, superior parietal lobule; STG, superior temporal gyrus.

## 4 DISCUSSION

We evaluated the variability of EEG electrode positions and their underlying brain regions using data recorded during a simultaneous EEG‐fMRI experiment. Overall, we found variance in electrode placement that was comparable with previous studies, with the largest deviations in the *z* dimension and in occipital and parietal electrodes. Consistent with previous findings, frontal electrodes had the smallest deviation across participants, in coordinates both at the scalp and projected onto the brain (Koessler et al., 2009; Okamoto et al., 2004). However, we did not identify any greater variation specifically in electrodes around the edge of the electrode cap, as previously found (Atcherson et al., 2007). We also did not find any consistent effect of cap size. However, as most participants required the average cap size of 56 cm, there were few data points from which to draw conclusions. In the future a more thorough examination of the influence of cap size on electrode position variability would therefore be beneficial. In addition, we outline a relatively simple pipeline for localizing electrodes using electrode gel artifacts, and provide the necessary analysis code for comparing scalp and cortex locations across participants.

These results have particularly important implications for studies using TMS. It is generally proposed that MRI‐guided stimulation is the most reliable approach to TMS (Bergmann & Hartwigsen, 2021; de Witte et al., 2018; Sack et al., 2009), and it is associated with increased disruptive effects (Beynel et al., 2019). However, it remains common practice to use the international 10‐10 and 10–20 layout systems to guide positioning for TMS stimulation, particularly when neuro‐navigation using structural or functional MRI scans is not possible (Beynel et al., 2019). This provides an approximate estimation of ROIs without the need for MRI scanning and will therefore be necessary for some experiments. Our results suggest that using EEG electrode position‐guided TMS may be more reliable for frontal electrodes, given the relatively small standard deviation found across participants. However, larger variation in the electrode position and underlying brain regions was found for electrodes at the back of the head, including occipital and parietal ROIs, which may feasibly lead to larger between‐subject differences in cortical stimulation with TMS.

Researchers also use the 10‐10 and 10–20 layout to inform electrode choice in EEG analysis. In accordance with previous results (Okamoto et al., 2004), electrode pairs C1/C2 and C3/C4 were not reliable for approximating the location of the motor cortex across participants. The mean locations of C3 and C4 were closer to the postcentral gyrus, and while neighboring electrode C1 was proximally located to the motor cortex (BA4), its pair electrode C2 was closer to the premotor cortex (BA6). Similarly, the mean location of electrode PO7 was mapped to BA18, whereas PO8 was closer to BA19. Cases such as these suggest that it may be beneficial to select the most relevant electrodes on an individual participant basis to calculate power or evoked potentials. Furthermore, source localization of EEG data is frequently used to provide an estimate of where in the brain a given change in electrical potential arises. However, interpreting source localization at the group level could be limited by the assumption that the relationship between electrode position and underlying cortical tissue is consistent across individuals (Dalal et al., 2014). Of course, electrical activity recorded at the level of the scalp is the summation of activity from multiple sources on the underlying cortex, and is not exclusively representing the neural activity in the closest region of the cortex (Nunez & Srinivasan, 2006). However, researchers generally select electrodes for analysis based on their proximity to the brain region of interest.

In addition to providing the results for one EEG‐fMRI dataset, we highlight a user‐friendly way of using electrode gel artifacts to localize electrode positions across participants. This pipeline takes advantage of existing functions in Brainsight; a software commonly used for neuro‐navigation in TMS, and therefore accessible for many neuroimaging centers. Although it is time consuming to manually label the position of each electrode for each participant, researchers could instead label a subset of electrodes for analysis (if not all are used). In this case, electrode positions were labeled after completion of the experiment. However, researchers can also use the functionality of Brainsight to mark the position of some/all electrodes on the EEG cap of each participant before beginning their experiment.

Our study is not without limitations. As this pipeline requires manual marking of electrode positions on the reconstructed scalp of the participant, error can be introduced by the subjective decision of the researcher. To combat this, every electrode position was checked and agreed on by both authors. A total of nine electrodes across five participants were re‐labeled during this checking procedure, all of which were more difficult to visualize given a very small or very large gel artifact. However, most positions were clearly visible on the Brainsight reconstruction, and the researchers agreed on the target locations of most electrodes. An additional source of variation could arise from the choice of atlas used for analysis. We used the Harvard‐Oxford Cortical Atlas to label the cortex underlying each electrode. The choice of atlas will influence the exact labeling, and we therefore chose a commonly used atlas available in FSL.

Finally, it is worth noting that participants were supine when structural scans were performed, rather than seated which is more common for EEG. It is possible that this altered the electrode positions, either through movement of the cap or through the redistribution of cerebrospinal fluid (Rice et al., 2013). However, during the original experiment the structural scans were the first to be acquired, limiting the amount of time during which participants could move from their initial position. Whilst it is possible that this still accounts for the greater variability in posterior electrode positions, our findings show a similar pattern to previous work (Koessler et al., 2009; Okamoto et al., 2004). Given that these studies used seated participants, it is unlikely that participant position can fully explain our results.

Overall, our results emphasize the variation in electrode positioning and underlying brain regions that can be found even using a fixed EEG cap, the latter most likely caused by between‐subject differences in brain morphology. Although these results are likely to vary across experiment and participant group, we provide an example case to demonstrate the potential variation in electrode positioning and underlying cortex across a sample group. We also outline a relatively simple pipeline for marking and analyzing the location of electrodes in a simultaneous EEG‐fMRI dataset with accompanying analysis code.

### PEER REVIEW

The peer review history for this article is available at https://publons.com/publon/10.1002/brb3.2476

## Supporting information

SUPPORTING INFORMATION

## Data Availability

We used 20 structural scans collected for a previously reported EEG‐fMRI experiment (Scrivener et al., 2021), for which the data are available at https://osf.io/w6bh3/. The secondary data for the current article, as well as MATLAB scripts used to analyze the data, are freely available at https://osf.io/853kw/.
