# Supplementary material for: Variability of EEG electrode positions and their underlying brain regions: visualizing gel artifacts from a simultaneous EEG‐fMRI dataset
Source: Brain Behav. 2022 Jan 18;12(2):e2476. doi: 10.1002/brb3.2476 (PMC8865144; doi:10.1002/brb3.2476)
Supplement: Supplementary file 1 — SUPPORTING INFORMATION [file BRB3-12-e2476-s001.docx]

**Supplementary Material:**

**Variability of EEG electrode positions and their underlying brain regions: visualising gel artifacts from a simultaneous EEG-fMRI dataset**

*Supplementary Table 1:* Skewness of the MNI coordinates for each electrode across participants, presented separately at the scalp and on the cortex. Skewness was calculated in Microsoft Excel.

|  | Scalp | | | Cortex | | |
| --- | --- | --- | --- | --- | --- | --- |
|  | x | y | z | x | y | z |
| AF3 | 0.66 | -0.07 | 0.09 | 0.55 | 0.02 | 0.02 |
| AF4 | 0.77 | -0.04 | 0.09 | 1.11 | 0.05 | 0.62 |
| AF7 | 0.83 | 0.54 | -0.33 | 0.50 | 0.67 | -0.37 |
| AF8 | 0.79 | -0.26 | 0.28 | 0.26 | 0.08 | 0.12 |
| AFZ | 0.32 | -0.07 | 0.42 | 0.40 | 0.13 | 0.41 |
| C1 | 0.03 | 0.80 | -0.13 | 0.14 | 0.79 | 0.32 |
| C2 | -0.63 | 0.28 | 1.01 | -0.42 | 0.19 | 1.17 |
| C3 | 0.09 | 1.06 | 0.19 | 0.06 | 0.98 | -0.01 |
| C4 | -0.26 | 0.01 | -0.03 | -0.14 | 0.11 | 0.56 |
| C5 | 0.27 | 1.46 | 0.23 | 0.10 | 1.32 | 0.14 |
| C6 | -0.24 | -0.24 | -0.14 | -0.53 | -0.03 | 0.14 |
| CP1 | -0.83 | 0.08 | -0.56 | -0.54 | 0.36 | -1.15 |
| CP2 | -0.76 | 0.19 | -0.12 | -0.31 | 0.15 | 0.01 |
| CP3 | -0.20 | 0.76 | -0.19 | -0.23 | 0.56 | -0.01 |
| CP4 | 0.47 | -0.22 | -0.39 | 0.85 | -0.47 | 0.72 |
| CP5 | 0.05 | 1.10 | 0.09 | 0.28 | 0.69 | -0.15 |
| CP6 | -0.03 | -0.08 | 0.08 | 0.21 | 0.09 | 0.04 |
| CPZ | -0.48 | 0.29 | 0.25 | -0.47 | 0.00 | 0.08 |
| CZ | -0.43 | 0.16 | 0.31 | -0.45 | -0.03 | 1.42 |
| F1 | 0.05 | 0.28 | -0.42 | 0.67 | 0.52 | 0.14 |
| F2 | 1.07 | 0.20 | 0.57 | 1.02 | 0.19 | 0.65 |
| F3 | 0.13 | 0.30 | -0.36 | 0.23 | 0.38 | -0.22 |
| F4 | 0.86 | 0.66 | 0.37 | 0.51 | 0.47 | 0.80 |
| F5 | 0.61 | 0.62 | 0.22 | 0.61 | 0.35 | 0.37 |
| F6 | 0.63 | 0.16 | 0.09 | -0.08 | 0.18 | 0.55 |
| F7 | 0.53 | 0.61 | -0.20 | 0.72 | 0.76 | -0.16 |
| F8 | 0.22 | -0.45 | 0.21 | -0.08 | -0.12 | 0.21 |
| FC1 | -0.16 | 0.83 | -0.93 | 0.36 | 0.89 | -0.22 |
| FC2 | 0.11 | 0.29 | 1.01 | 0.12 | 0.43 | 1.52 |
| FC3 | 0.30 | 0.31 | 0.02 | 0.47 | 0.41 | -0.10 |
| FC4 | 0.06 | 0.31 | 0.53 | -0.02 | 0.10 | 0.84 |
| FC5 | -0.29 | 0.80 | 0.77 | 0.76 | 0.53 | 0.20 |
| FC6 | 0.04 | -0.34 | -0.07 | -0.25 | -0.13 | 0.27 |
| FCZ | 0.21 | 0.71 | 0.26 | 0.30 | 0.65 | 1.07 |
| FP1 | 1.11 | 0.59 | -0.11 | 1.07 | -0.38 | -0.21 |
| FP2 | 0.91 | -0.37 | 0.60 | 1.10 | 0.27 | 0.52 |
| FPZ | 0.61 | 0.32 | 0.11 | 0.65 | 0.32 | 0.23 |
| FT10 | -0.47 | -0.12 | 0.13 | 0.12 | -0.52 | 0.23 |
| FT7 | 1.29 | 1.10 | 0.54 | 0.87 | 1.12 | 0.42 |
| FT8 | -0.09 | -0.61 | -0.11 | 0.09 | -0.58 | -0.20 |
| FT9 | 0.22 | 0.29 | 1.61 | -0.09 | 0.34 | 1.44 |
| FZ | 0.31 | 0.32 | -0.25 | 0.09 | 0.41 | -0.09 |
| O1 | -0.70 | 0.21 | -0.30 | -0.69 | 0.12 | -0.38 |
| O2 | -0.77 | 0.44 | -0.26 | -0.86 | 1.06 | -0.45 |
| OZ | -0.68 | 0.96 | -0.38 | -0.76 | 0.37 | -0.33 |
| P1 | 0.11 | 0.57 | -0.12 | 0.24 | -0.13 | -0.16 |
| P2 | 0.21 | 0.21 | -0.04 | 0.61 | 0.29 | 0.57 |
| P3 | -0.50 | 1.09 | -0.26 | -0.51 | 0.44 | -0.35 |
| P4 | 0.34 | -0.12 | 0.07 | 0.75 | -0.21 | 0.06 |
| P5 | -0.18 | 1.24 | 0.18 | 0.19 | 0.76 | 0.01 |
| P6 | -0.19 | -0.26 | -0.09 | 0.32 | 0.10 | -0.15 |
| P7 | -0.91 | 1.06 | 0.39 | -0.45 | 0.69 | 0.37 |
| P8 | -0.31 | -0.23 | -0.05 | -0.39 | 0.43 | 0.00 |
| PO3 | -0.57 | 0.67 | -0.13 | -0.85 | -0.09 | 0.03 |
| PO4 | -0.13 | 0.54 | 0.05 | 0.02 | 0.28 | -0.14 |
| PO7 | -0.64 | 1.24 | -0.04 | -0.98 | 0.85 | -0.02 |
| PO8 | -0.82 | -0.68 | -0.13 | -0.69 | -0.37 | -0.25 |
| POZ | -0.26 | 0.31 | -0.05 | -0.09 | 0.23 | -0.07 |
| PZ | -0.07 | 0.23 | -0.16 | 0.08 | 0.01 | 0.21 |
| T7 | 1.09 | 1.44 | 0.78 | 0.15 | 1.07 | 1.03 |
| T8 | 0.49 | -0.15 | -0.09 | -0.37 | 0.01 | -0.06 |
| TP7 | -0.97 | 1.43 | 0.56 | 0.25 | 1.49 | 0.64 |
| TP8 | 0.01 | -0.11 | 0.21 | -0.23 | 0.03 | 0.39 |
| TP9 | 0.08 | -0.50 | 0.96 | 0.30 | -0.40 | 0.69 |
| TP10 | -0.86 | -0.05 | 0.00 | -0.36 | -0.61 | 0.13 |
